# Laparoscopy shows superiority over endoscopy for early detection of malignant atrophic papulosis gastrointestinal complications: a case report and review of literature

**DOI:** 10.1186/s12876-015-0387-y

**Published:** 2015-11-02

**Authors:** A. E. Toledo, L. S. Shapiro, J. F. Farrell, C. M. Magro, J. Polito

**Affiliations:** 1 Steffens Scleroderma Center, Saratoga Springs, NY USA; 2 The Center for Rheumatology, Albany and Saratoga Springs, NY USA; 3 Albany College of Pharmacy and Health Sciences, Albany, NY USA; 4 Weill Cornell College of Cornell University, New York, NY USA; 5 Gastroenterology Consultants, Albany, NY USA

**Keywords:** MAP, Malignant, Atrophic papulosis, Treprostinil, Eculizumab, Laparoscopy

## Abstract

**Background:**

The malignant form of atrophic papulosis (Köhlmeier-Degos disease) is a rare thrombo-occlusive vasculopathy that can affect multiple organ systems. Patients typically present with distinctive skin lesions reflective of vascular drop out. The small bowel is the most common internal organ involved, resulting in considerable morbidity and mortality attributable to ischemic microperforations. Determination of the presence of gastrointestinal lesions is critical in distinguishing systemic from the benign, cutaneous only disease and in identifying candidates for treatment.

**Case presentation:**

We describe an 18 year old male who first presented with cutaneous atrophic papulosis but became critically ill from small bowel microperforations. He had an almost immediate and dramatic response to treatment. Prior to his presentation with acute abdomen he had upper and lower endoscopy showing areas of nonspecific patchy erythema. At laparotomy, innumerable characteristic lesions with central pearly hue and erythematous border were seen. PubMed was used for a literature search using the keywords malignant atrophic papulosis, Degos disease, endoscopy, laparoscopy and laparotomy. This search yielded 200 articles which were further analyzed for diagnostic procedures and findings.

Among the 200 articles we identified only 11 cases in which endoscopy was performed. Results of endoscopy and laparotomy in our patient with malignant atrophic papulosis were compared to those in the literature. Endoscopy of the gastrointestinal tract has shown gastritis and non-specific inflammation whereas laparoscopy shows white plaques with red borders on the serosal surface of the small bowel and the peritoneum. From personal communications with other physicians worldwide, we identified three additional unpublished cases in which endoscopy revealed only minimal changes while laparoscopy showed dramatic lesions. From our experience the endoscopic findings are often subtle and nonspecific, whereas laparascopy or laparotomy will reveal pathognomic lesions on the serosal surface of the intestine.

**Conclusion:**

Our report contrasts the endoscopic and laparoscopic findings in malignant atrophic papulosis which suggest laparoscopy is the more powerful means of detecting gastrointestinal involvement. Imaging studies may serve as a key indicator of systemic progression. Based on our experience, laparoscopy should be performed when there is a high index of suspicion for gastrointestinal malignant atrophic papulosis, even if endoscopic examination is non-diagnostic or normal.

**Electronic supplementary material:**

The online version of this article (doi:10.1186/s12876-015-0387-y) contains supplementary material, which is available to authorized users.

## Background

Atrophic papulosis (Köhlmeier-Degos disease) is a rare thrombo-occlusive vasculopathy that can affect multiple organ systems. Patients primarily present with distinctive skin lesions reflective of vascular drop out. The small bowel is the most common internal organ involved, resulting in considerable morbidity and mortality attributable to ischemic microperforations [1–3]. Skin lesions associated with atrophic papulosis appear as avascular, slightly depressed porcelain-like lesions with a telangiectatic rim [3]. The more common cutaneous manifestations of a primary vasculopathy, ulceration, livedo and palpable purpura, are not seen. Gastrointestinal (GI) involvement develops approximately 60 % of the time, usually one to three years after the skin lesions first appear [4]. The skin and GI lesions show striking similarities on gross examination even though the caliber of the affected vessels is different. In the skin, the targeted vessels are capillaries and venules of the dermis, whereas in the gastrointestinal tract subserosal arteries and arterioles are the primary target. While both vascular lesions result in a similar depressed porcelain scar, the nature of the vasculopathy histologically is different. In the skin the primary vascular pattern is a thrombogenic microangiopathy while in the gut, an obliterative mucinous fibrointimal arteriopathy is seen [4, 5]. Until recently, reported cases of systemic disease have been descriptions of an inexorable path to a fatal outcome [2]. Reports of prominent C5b-9 deposits in skin and gastrointestinal tissue of patients with the malignant form of atrophic papulosis (MAP) have led to the use of eculizumab, an inhibitor of C5 activation, with prolonged survival in some cases [1]. Additionally, a recent report suggests the efficacy of treprostinil as adjunctive therapy in patients with MAP [2].

New hypotheses to the pathogenesis of MAP suggests two forms of vasculopathy which are mechanistically distinctive and have helped identify new treatment options. One aspect is a thrombotic microangiopathy attributable to endothelial cell injury. Prior studies have shown extensive vascular deposits of C5b-9 as the basis of injury analogous to other catastrophic C5b-9 mediated microvascular injury syndromes such as atypical hemolytic uremic syndrome [6]. Eculizumab has an almost immediate effect, preventing further formation and deposition of the membrane attack complex, thereby decreasing C5b-9 stimulated apoptosis. It also immediately inhibits formation of the anphylatoxin C5a [1]. This particular microangiopathy is also associated with a type I interferon rich microenvironment as revealed by extensive vascular deposition of myxovirus protein, a surrogate marker of type I interferons [6, 7]. Deficiency of endothelial progenitor cells (EPC) is one hypothesis in the pathogenesis of MAP. EPC exposed to interferon-alpha induce the up-regulation of IL-18 and caspase-1. IL-18 inhibits vascular repair while caspase-1 contributes to EPC dysfunction. In essence there is an imbalance between vascular damage and vascular repair. Eculizumab has no effect on the interferon-mediated injury. Recent data suggest treprostinil, a prostacyclin analog that facilitates healing of ischemic tissue and inhibits platelet aggregation, could ameliorate symptoms and signs of MAP by virtue of its effect on enhancing EPC repair [1, 2, 8]. Because of this, treprostinil has been employed as adjunctive therapy in patients with MAP [9].

Determination of the presence of gastrointestinal lesions is critical. Untreated, individuals with such lesions will have progressive disease culminating in repeated bowel perforations and death. At present, there is no consensus on the best means of detecting gastrointestinal lesions in patients with cutaneous atrophic papulosis.

## Case presentation

We describe an 18 year old male who presented with cutaneous atrophic papulosis but became critically ill from small bowel microperforations. We also describe his sister, who developed similar skin lesions compatible with a familial variant. PubMed was used for a literature search using the keywords malignant atrophic papulosis, Degos disease, endoscopy, laparoscopy and laparotomy. The search yielded 200 articles which were further analyzed for diagnostic procedures and findings. Also personal communications with other physicians caring for individuals with systemic MAP led to evaluation and comparison of gastrointestinal imaging modalities.

### Patient One:

**Fig. 1:**
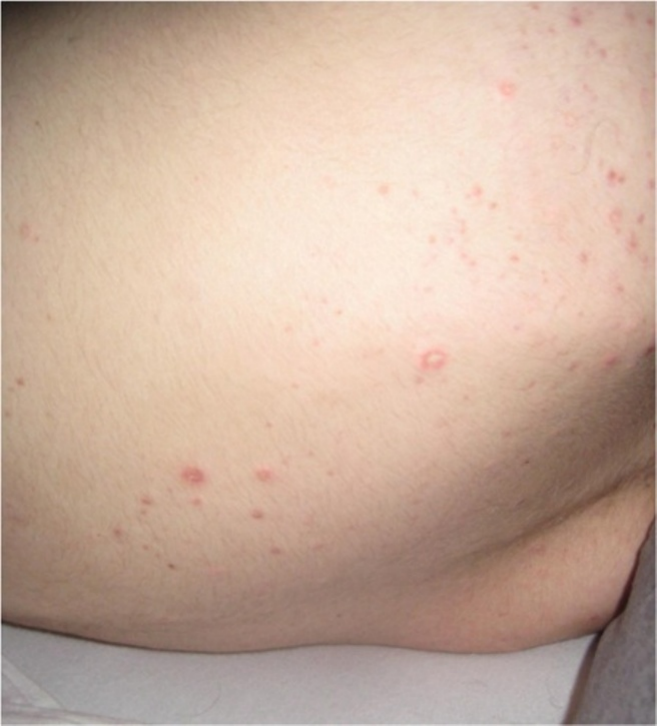
Patient one’s rapidly proliferating skin lesions

**Fig. 2:**
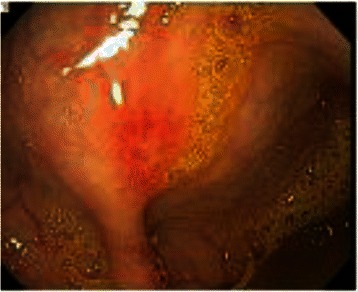
Patient one, lower endoscopy showed patchy erythematous mucosa with central clearing at the sigmoid, rectosigmoid colon and splenic flexure

**Fig. 3:**
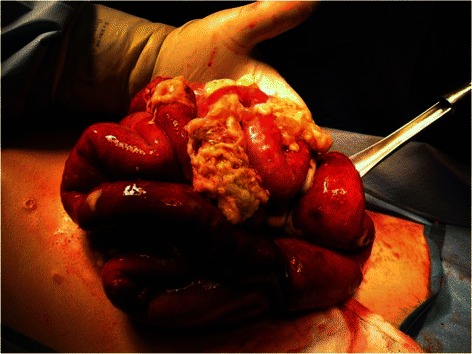
Patient one, at laparotomy, innumerable typical lesions of 0.3–0.5 cm papules with central pearly hue and erythematous border were seen

Patient One is an 18 year old male who presented with cutaneous disease, had rapidly proliferating skin lesions over a course of a year, and then survived small bowel microperforations from MAP (Fig. 1). Two months prior to his presentation with acute abdomen, lower endoscopy showed patchy erythematous mucosa with central clearing at the sigmoid, rectosigmoid colon and splenic flexure (Fig. 2). Upper endoscopy demonstrated mild acute gastritis. At laparotomy, innumerable typical lesions of 0.3-0.5 cm papules with central pearly hue and erythematous border were seen (Fig. 3). The patient had a very rapid and dramatic response to therapy with eculizumab [10]. Later on the patient required a second agent, treprostinil, for maintenance and suppression of other systemic manifestations. Five years have passed and he has been doing well on double therapy of eculizumab and treprostinil.

### Patient Two:

Patient Two is the sister of Patient One. She slowly developed a small number of cutaneous lesions that were biopsied and were consistent with atrophic papulosis. She had minor gastrointestinal discomfort. For this reason and due to her brother's history a laparoscopy was performed with normal findings. Her abdominal discomfort spontaneously resolved. Given the lack of GI findings and the highly limited nature of the cutaneous presentation she is less likely to progress to systemic MAP.

### Personal communications

#### Patient A:

**Fig. 4:**
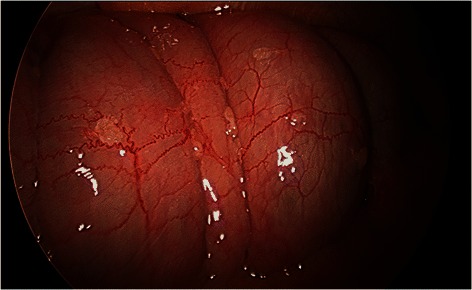
Patient A, colonoscopy was normal but exploratory laparoscopy revealed innumerable MAP lesions

A 44 year old female with cutaneous lesions of atrophic papulosis developed sudden monocular vision loss and severe eye pain requiring enucleation with pathologic findings of microvascular changes in the orbit consistent with MAP. Colonoscopy in April 2012 showed normal findings but exploratory laparoscopy in October 2012 revealed innumerable MAP lesions (Fig. 4).

#### Patient B:

A 36 year old male with MAP overlapping with dermatomyositis had a colonoscopy showing only nonspecific ischemic colitis and a CT scan demonstrated colitis and enteritis but then developed microperforations with lesions typical of MAP noted at time of surgery [11].

#### Patient C:

A patient in Malaysia had nonspecific endoscopy and normal colonoscopy but striking laparoscopic changes.

#### Patient D:

**Fig. 5:**
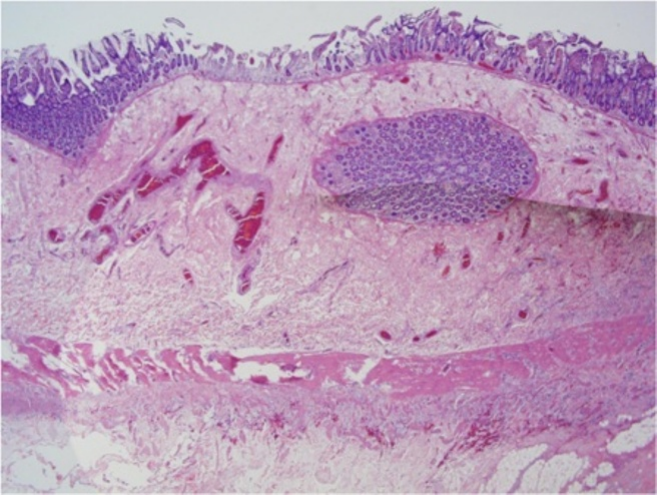
This low power view shows a relatively normal appearing colonic mucosa however the subserosa appears very edematous

**Fig. 6:**
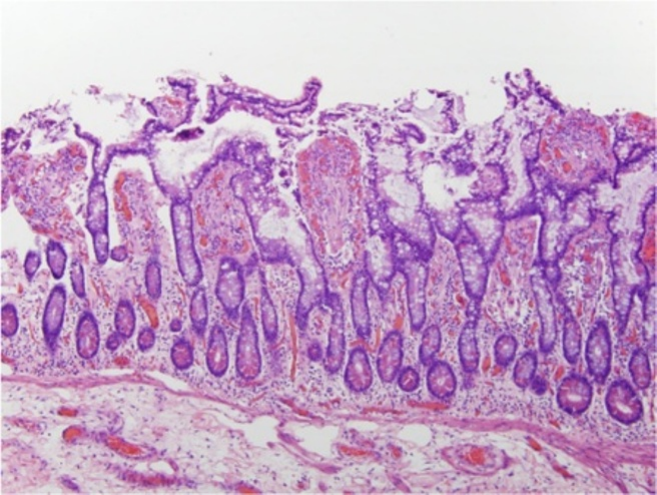
Higher power magnification demonstrates a normal appearing colonic mucosa despite severe inflammatory and vascular pathology involving the serosal surface and subserosa

**Fig. 7:**
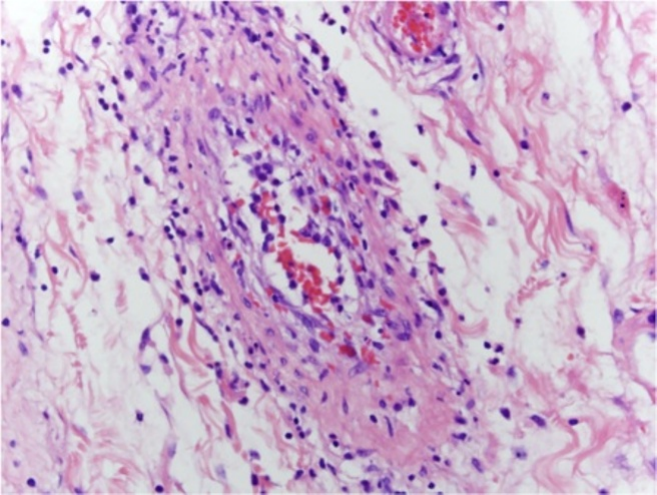
Within the subserosa and to a lesser extent the submucosa, an arteriopathy exhibiting varying stages of inflammation and fibro-obliterative healing is identified. In this photomicrograph, the endothelium appears activated and is detached from the intima compatible with primary endothelial cell injury. As part of the acute response, there is an endoluminal influx of inflammatory cells especially macrophages. Note the red cell engulgment, a finding indicative of macrophage activation

**Fig. 8:**
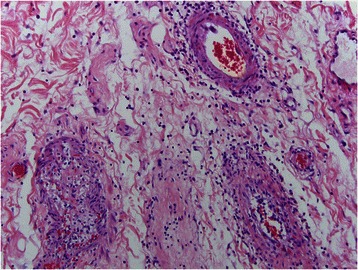
In this subserosal vessel, there is still the residuum of an inflammatory endothelialitis however there is progressive intimal fibrosis

**Fig. 9:**
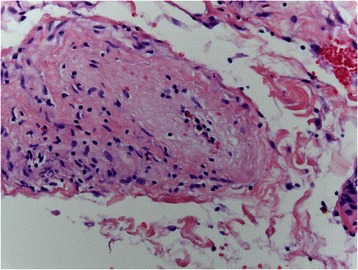
In this subserosal vessel the vascular lumen is now occluded by collagen defining the end stage fibrous obliterative lesion of Degos disease. The subsequent ischemia leads to compromise of the bowel, eventuating in transmural infarction. The latter reflects the upstream effects of the subserosal larger vessel arteriopathy to the microcirculation of the remainder of the bowel

A previously reported 43 year old male patient had a normal CT scan and repetitive endoscopic biopsies and a colonoscopy that were unremarkable but after development of acute abdomen, a resected portion of bowel showed extensive subserosal fibrobliterative arteriopathic and thrombotic microvascular disease diagnostic of MAP [1] (Figs. 5, 6, 7, 8, 9).

#### Patient E:

This previously reported patient was 36 years old male with dermatomyositis who presented with classic skin lesions of atrophic papulosis. He then developed severe gastrointestinal symptoms. While endoscopy and CT scan were unremarkable, laparoscopy revealed classic lesions of MAP. The patient died of sepsis [12].

Among the 200 cases that were reviewed we found 11 in which endoscopy was performed. Results of endoscopy and laparotomy in our patients with MAP were analyzed and compared to those in the literature. Based on the published cases, 9 out of the 11 endoscopies performed had non-specific endoscopic changes either prior to or near-simultaneous to catastrophic bowel perforation [9, 10, 13]. Laparotomy findings are striking and predictably present in those with acute abdomen. In the cases found in the literature and in our Patient One, endoscopic evaluation revealed pearly-rose patches with a surrounding hyperemic rim in the mucosa which may be interpreted as general inflammation. Findings are most commonly seen in the small bowel, especially jejunum, but can involve any part of the gastrointestinal lumen. More pathognomic findings are found at laparoscopy, and are characterized by multiple white plaques with red borders involving the serosal surface of the small bowel and the peritoneum [14, 15]. Laparoscopic lesions are similar to the skin lesions seen in patients with MAP [10]. The gross serosal porcelain plaques in concert with the normal endoscopic findings are well exemplified by the pathology of resection specimens in patients with MAP. In particular in patients with an acute abdomen necessitating bowel resection, the cardinal hallmarks are an intact mucosa with mild inflammation or no inflammation in concert with striking obliterative arteriopathic changes most conspicuous in subserosal vessels but also showing variable involvement within the submucosal. Concomitant subserosal inflammation and edema may be seen. The end sequelae is an ischemic one characterized by a severe necrotizing serositis.

## Conclusions

Historically, death in patients with MAP has most commonly resulted from gastrointestinal microperforations and subsequent sepsis. Our experience successfully treating Patient One led us to investigate means of early detection of gastrointestinal involvement. Early intervention with effective therapy could prevent progression to bowel perforation. At present, available therapies are most often initiated as an act of desperation in patients who are already critically ill with advanced multiorgan failure.

Extensive and rapidly progressive skin lesions are more likely to be associated with the early development of systemic disease. We believe any such individual, even if asymptomatic, and any individual with cutaneous atrophic papulosis and unexplained abdominal pain should undergo laparoscopy as the best means of detecting gastrointestinal involvement.

Due to the rarity of MAP, diagnostic guidelines were only recently presented [3], while therapeutic ones do not exist. When patients present with acute abdomen, findings at laparotomy or laparoscopy are always striking. Previously published cases of MAP have reported time to death after detection of systemic disease to be one to two years [3, 16]. Patient One, however, is currently a 5 year survivor of severe systemic MAP. With the evolution of therapies that have altered the previously grim prognosis, pursuing early detection of systemic disease is essential in more consistently improving disease outcome.

Endoscopic evaluation has been suggested for all patients with classic skin lesions of atrophic papulosis with specific or nonspecific symptomatology on the central or peripheral nervous system, gastrointestinal tract or any other organ system [3]. Unfortunately since the jejunum is the most commonly involved section of the gastrointestinal tract and because the vascular lesion is primarily subserosal and not mucosal [4], endoscopic evaluation can and has given false negative results. Capsule endoscopy can be added to the workup to provide additional imaging of the jejunum but, again, the view is mucosal and the pathology is subserosal. Our experience suggests laparoscopy is a more sensitive diagnostic tool that should be implemented on higher risk patients [13]. High risk patients include those whose skin lesions are proliferating rapidly and any with unexplained gastrointestinal symptoms [3, 17–19]. CT scanning of the abdomen is also an insensitive method of detection of enteric involvement of MAP [20]. Laparoscopy may uncover systemic involvement in an otherwise asymptomatic patient with only cutaneous lesions [21]. MAP like many autoinflammatory conditions is a targeted organ and site specific disease exhibiting a peculiar and distinctive predilection to involve select small arteries and arterioles of the subserosa with relative sparing of the microvasculature of the lamina propria.

After 5 years of close observation of Patient One we believe we have identified strategies for early detection with laparoscopy as a potential lifesaving tool. GI involvement with symptoms such as dyspepsia, abdominal pain, nausea, vomiting and others should be further investigated in MAP patients. While the gastrointestinal tract can be examined by endoscopy, to look for white, yellowish, or pearly-rose patches with a hyperemic rim [14] the changes may be subtle and can be easily missed using this method [13, 18]. Laparoscopy can more effectively identify the diagnostic lesions of gastrointestinal MAP [13, 14]. Laparoscopic identification of white spots with hyperemic borders on the serosal surface of the bowel and the peritoneum are diagnostic of MAP and do not require a biopsy. Their identification should warrant immediate treatment [1, 2, 9, 13]. From our literature search we do not recommend serosal biopsy of the lesions since it can lead to seromuscular breach [4, 22].

These findings can help patients obtain faster and easier access to treatments as they emerge. Since clear diagnostic and treatment guidelines for MAP do not exist and untreated systemic MAP has a fatal prognosis, there is need to identify patients at risk for GI microperforations, sepsis, and death. Our experience and the other cases we report such that visualization of the serosa of the bowel and peritoneum by laparoscopy is superior to endoscopic evaluation. Prospective studies are necessary to clarify the role of both endoscopy and laparoscopy in the detection of systemic disease in patients with cutaneous atrophic papulosis and to establish the frequency with which such studies should be repeated in those in whom the findings are negative.

## Consent

Written informed consent was obtained from all patients (and guardians) for publication of this case report and any accompanying images. A copy of the written consent is available for review by the Editor of this journal.

## Additional file

Additional file 1: **CARE Checklist (2013) of information to include when writing a case report.** (DOCX 1483 kb)

## Abbreviations

MAP: Malignant atrophic papulosis
GI: Gastrointestinal
EPC: Endothelial progenitor cells
